# Designing Artificial Laccase Catalysts by Introducing Substrate Oxidation Metals into Oxygen‐Reducing Metal‐Organic Frameworks: Cu‐Doped ZIF‐67

**DOI:** 10.1002/chem.202402953

**Published:** 2024-11-16

**Authors:** Hiroki Nakahara, Yutaka Hitomi

**Affiliations:** ^1^ Department of Applied Chemistry Graduate School of Science and Engineering Doshisha University 1-3 Tatara Miyakodani 610-0321 Kyotanabe, Kyoto Japan

**Keywords:** Laccase, Copper, Cobalt, Enzyme model, Redox chemistry

## Abstract

Laccase, a multi‐copper oxidase, is limited by its optimal temperature range and isolation costs. To overcome these challenges, we synthesized copper‐doped zeolitic imidazolate framework‐67 (Cu‐doped ZIF‐67) with 16 mol % Cu as an artificial laccase catalyst. The introduced Cu site acts as the phenol oxidation site, and Co‐based ZIF‐67 is the four‐electron oxygen reduction site. Laccase also employs this division of oxidation and reduction sites. Cu‐doped ZIF‐67 demonstrated significant catalytic activity, superior to natural laccase, especially at elevated temperatures, and maintained stability across multiple reaction cycles. These findings suggest that Cu‐doped ZIF‐67 is a robust, reusable alternative for industrial applications requiring high thermal stability and efficient catalysis.

## Graphical abstract

This study presents the development of copper‐doped zeolitic imidazolate framework‐67 (Cu‐doped ZIF‐67) as an artificial laccase catalyst. Cu‐doped ZIF‐67 exhibits enhanced catalytic performance and thermal stability compared to natural laccase, providing an efficient and reusable solution for industrial applications. The dual‐site mechanism, where copper facilitates phenol oxidation and cobalt‐based ZIF‐67 supports oxygen reduction, is thoroughly explored.

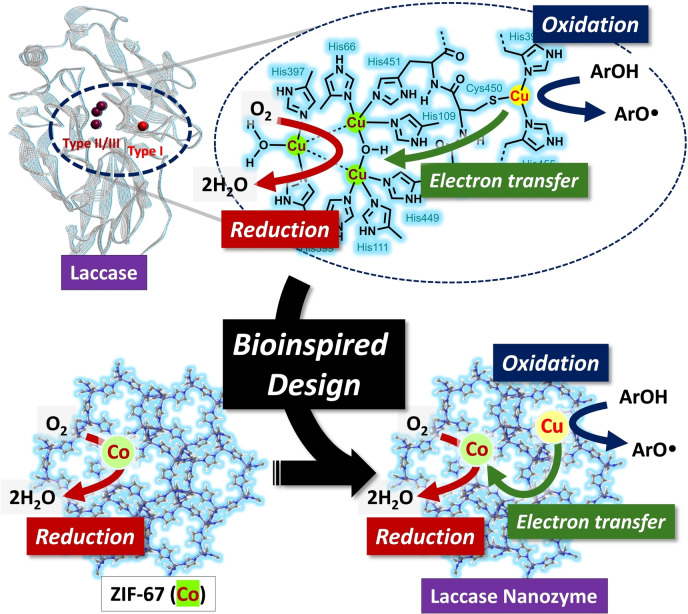

## Introduction

Laccase is a multi‐copper oxidase that oxidizes phenols using molecular oxygen as an oxidant. Applications of laccase include bioremediation of environmental pollutants, decolorization of dyes in the textile industry, modification of lignin in the pulp and paper industry, stabilization of beverages such as wine and fruit juices in the food industry, hair dye products, biosensors for various detection purposes, and the organic synthesis of pharmaceuticals.[^1^, ^2^, ^3^, ^4^, ^5^, ^6^, ^7^, ^8^]

The optimal temperature of laccase varies depending on the type of enzyme. Generally, laccases exhibit activity in the range of 30–50 °C, but some thermostable laccases also exist. A novel laccase isolated from *Bacillus pumilus* TCCC 11568 shows activity at 80 °C, but its activity decreases by half within two hours. Additionally, the isolation and purification of laccase are costly, limiting its utilization.^[9]^

Research and development of laccase mimics have been conducted to address these issues.[^1^, ^10^, ^11^, ^12^, ^13^, ^14^, ^15^, ^16^, ^17^, ^18^] To date, various copper‐based complexes have been reported to show the ability to catalyze aerobic oxidation reactions similar to those performed by laccase enzymes. Besides discrete copper complexes, materials containing multiple copper ions have also been developed as laccase mimics. For example, Wang *et al*. reported that a Cu‐based MOF, prepared from copper ions and 5‐(sulfomethyl) isophthalic acid, exhibited a lower *K*_m_ value compared to both laccase and other reported laccase mimetics, with a detection limit of 0.53 μM for 2,4‐dinitrophenol (2,4‐DP), one of the phenolic pollutants.^[17]^ In all previously reported Cu‐based MOFs with laccase‐like activity, the copper ions in the same environment are responsible for both the oxidation of phenolic substrates and the reduction of oxygen.

Another approach involves stabilizing laccase by encapsulating it in metal‐organic frameworks (MOFs).[^19^, ^20^] Encapsulating laccase in MOFs enhances its thermal stability. For instance, when laccase is encapsulated in ZIF‐8 (ZIF: zeolitic imidazolate framework), its stability increases at temperatures up to 70 °C. Laccase encapsulated in MOFs also shows resistance to organic solvents such as *N*,*N*‐dimethylformamide.^[21]^ Moreover, laccase immobilized in MOFs maintains activity over multiple reaction cycles. For example, laccase immobilized in Fe‐doped ZIF‐8 retains 59 % of its activity after five reaction cycles.^[22]^ Thus, these are remarkable achievements, but they do not solve the problem of laccase purification costs.

**Figure 1:**
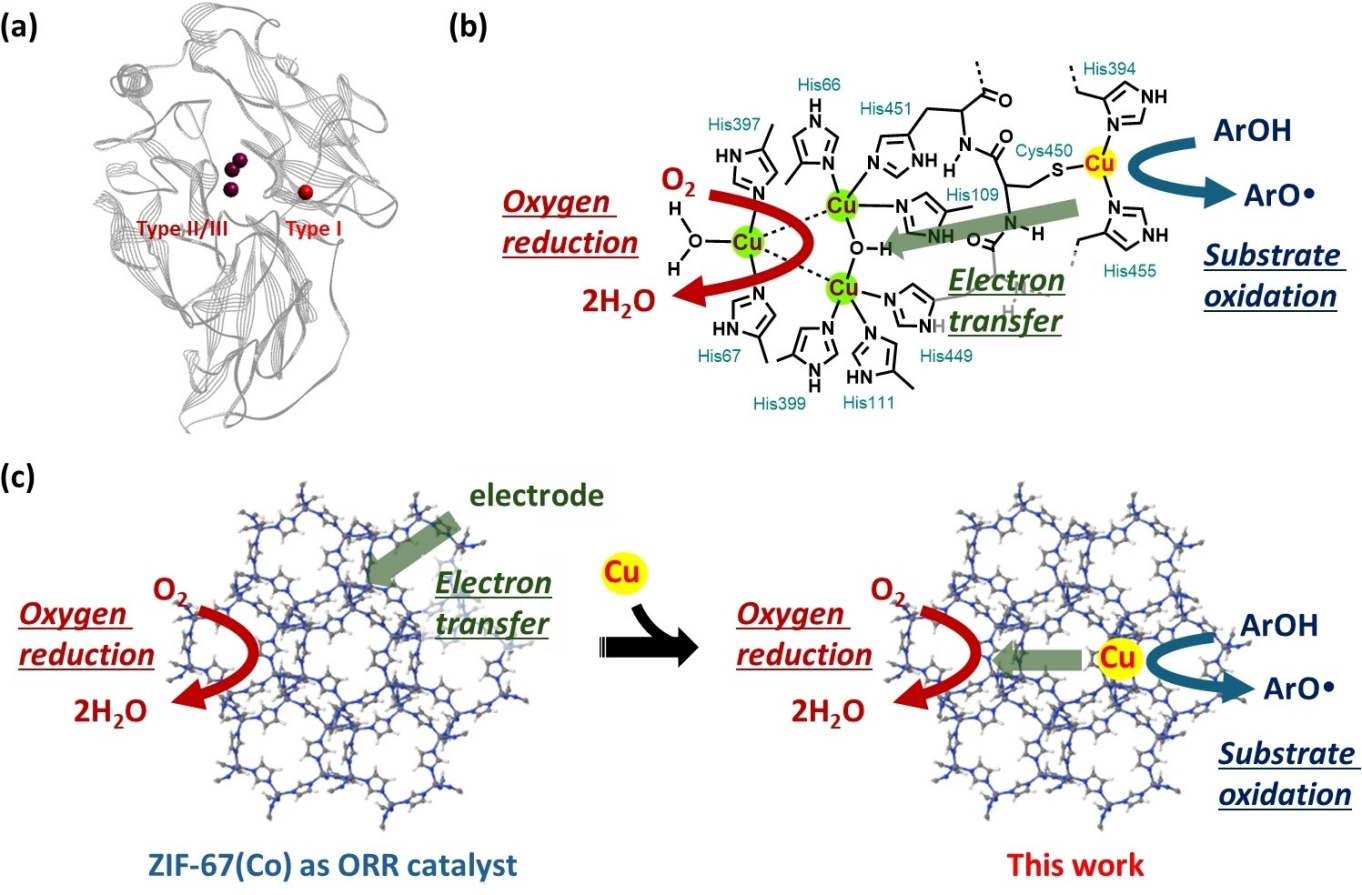
(a) Cartoon representation of the crystal structure of the laccase (PDB 5ANH)^[23]^ with the four coppers depicted as orange spheres. (b) A zoom‐up of the active site. (c) The design concept of an artificial laccase catalyst, Cu‐doped ZIF‐67.

Laccase possesses a mononuclear copper site (Type I) responsible for the one‐electron oxidation of phenolic substrates and a trinuclear copper cluster (Type II and Type III) responsible for the four‐electron reduction of oxygen (Figure 1a and b).[^6^, ^23^] The electrons generated by oxidizing substrates at the Type I copper site are transferred to the trinuclear copper cluster (Type II/III) near the Type I copper site and used in the four‐electron reduction of oxygen. This electron transfer‐mediated coupling of substrate oxidation and oxygen reduction is the unique strategy behind laccase's high activity. However, no artificial systems have successfully replicated laccase's strategy of separating the substrate oxidation site from the oxygen reduction site to achieve laccase‐like acitivty.

ZIF‐67, a metal‐organic framework composed of cobalt ions and 2‐methylimidazole, has been reported as a catalyst for the four‐electron reduction of oxygen to water.[^24^, ^25^] Several reports detail the use of ZIF‐67 and its derivatives as precursors for oxygen reduction catalysts, primarily in electrochemical oxygen reduction. When pyrolyzed or treated under specific conditions, it forms catalytically active materials for the oxygen reduction reaction (ORR). However, ZIF‐67 itself acts as an ORR catalyst (Figure 1c, left).

In this study, we hypothesize that by substituting some cobalt ions in ZIF‐67 with copper ions, the copper sites can act as substrate oxidation sites similar to laccase's Type I copper site, while the remaining cobalt sites serve as ORR catalysts (Figure 1c, right). Furthermore, if these oxidation and reduction sites are coupled through electron transfer reactions, Cu‐doped ZIF‐67 is expected to exhibit superior laccase‐like activity.

## Results and Discussion

Cu‐doped ZIF‐67 was synthesized following the previously reported synthesis method for Cu‐doped ZIF‐8, where ZIF‐8 is the zinc counterpart of ZIF‐67.^[26]^ When synthesizing Cu‐doped ZIF‐67 using equimolar amounts of Cu(NO_3_)_2_ and Co(NO_3_)_2_, referred to hereafter as Cu50‐ZIF‐67, inductively coupled plasma atomic emission spectroscopy (ICP‐AES) measurements revealed that only 16 mol % of the total metal ions were Cu. As we previously reported, when Cu‐doped ZIF‐8 was synthesized using 50/50 mole ratios of Cu(NO_3_)_2_ and Zn(NO_3_)_2_, 40 mol % of the total metal ions were Cu.^[26]^ The lower than 50 % content of Cu of Cu‐doped ZIF‐67 indicates that Cu is less readily incorporated into Co‐based ZIF‐67 than Zn‐based ZIF‐8. Cu(II) ions prefer planar tetradentate coordination and have an ionic radius of 71 pm, while Zn has an ionic radius of 74 pm and Co has an ionic radius of 72 pm. Thus, the ionic radii of Cu and Co are closer than those of Cu and Zn. Therefore, the difference in the incorporation of Cu ions between Cu‐doped ZIF‐8 and Cu‐doped ZIF‐67 cannot be attributed solely to the difference in ionic radii. It is suggested that Co(II) ions, being more inert to ligand exchange compared to Zn(II) ions, form the ZIF structure predominantly with Co(II), thereby excluding the less readily incorporated Cu(II) ions. Given the limited incorporation of Cu ions, it is unsurprising that the XRD, diffuse reflectance spectra, and particle shape (as determined by SEM image analysis) of Cu‐doped ZIF‐67 showed little change compared to ZIF‐67 (Figures S1–S3).

In addition to Cu50‐ZIF‐67 (16 mol % Cu), we prepared Cu25‐ZIF‐67 and Cu75‐ZIF‐67 with adjusted Cu/Co ratios of 25/75 and 75/25, respectively. However, the yield of Cu75‐ZIF‐67 was only 5.5 %, four times lower than that of Cu50‐ZIF‐67. Unexpectedly, Cu75‐ZIF‐67 contained only ten mol % of Cu, lower than the Cu content in Cu50‐ZIF‐67 (16 mol % Cu). While the exact reason for this is unclear, due to the low yield of Cu75‐ZIF‐67, we decided to use Cu25‐ZIF‐67 (7 mol % Cu) and Cu50‐ZIF‐67 (16 mol % Cu) in subsequent experiments.

**Scheme 1:**
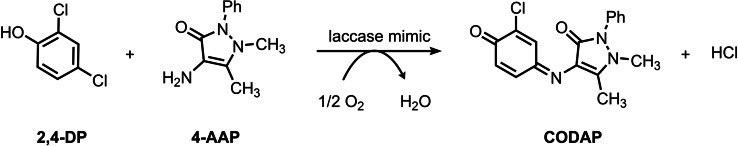
Reaction for Colorimetric Assay of Laccase‐like Activity.

**Figure 2:**
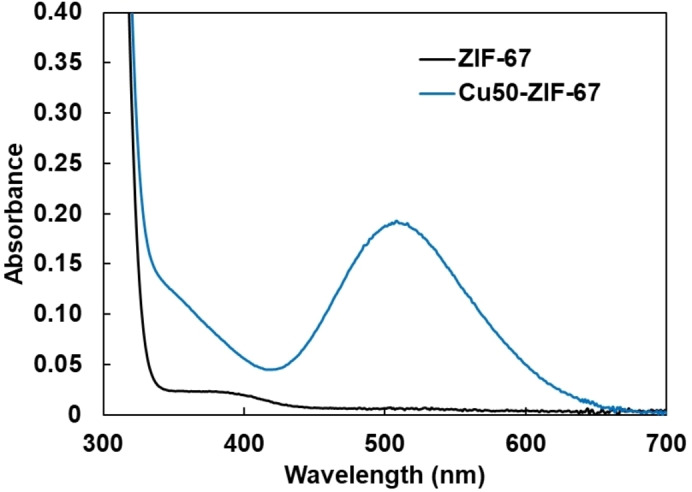
Absorption spectra of the colorimetric assay using Cu50‐ZIF‐67 (blue) and ZIF‐67 (black) at 300 seconds after reaction initiation, each at a metal concentration of 200 μM.

**Figure 3:**
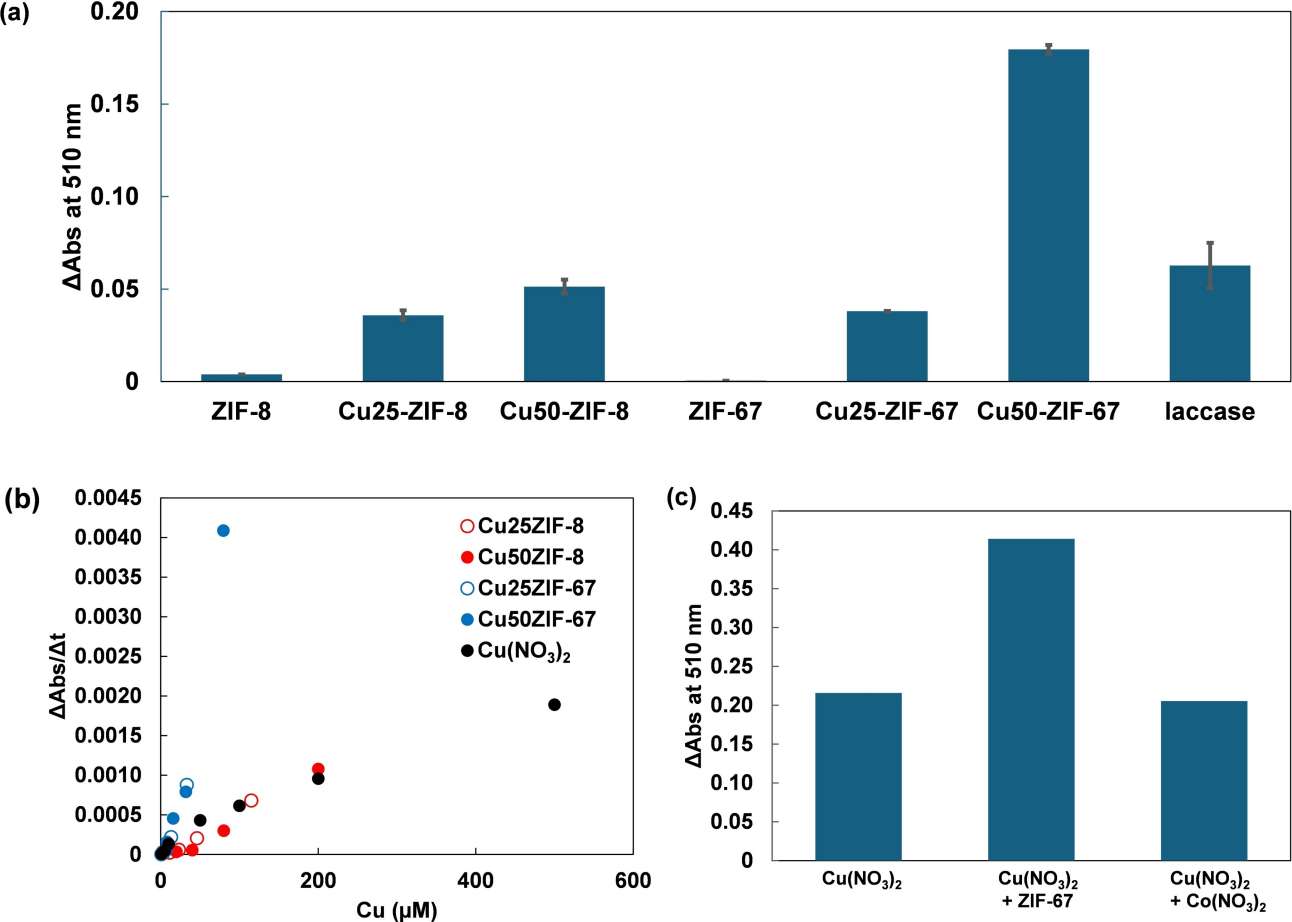
(a) Bar graph of absorbance at 510 nm after 300 seconds for the colorimetric assay using catalysts: ZIF‐67, Cu50‐ZIF‐67 (16 mol % Cu), Cu25‐ZIF‐67 (7 mol % Cu), ZIF‐8, Cu50‐ZIF‐8 (40 mol % Cu), each at a metal concentration of 200 μM, and laccase ([Cu]=200 μM). Error bars represent data from three replicates. (b) Plot of the initial increase rate of absorbance intensity at 510 nm for the colorimetric assay using catalysts: Cu50‐ZIF‐67 (16 mol % Cu), Cu25‐ZIF‐67 (7 mol % Cu), Cu50‐ZIF‐8 (40 mol % Cu), Cu25‐ZIF‐8 (23 mol % Cu), and Cu(NO_3_)_2_ as a function of copper concentration. (c) Bar graph showing absorbance at 510 nm after 300 seconds for the colorimetric assay using different catalysts: Cu(NO_3_)_2_ alone (100 μM), Cu(NO_3_)_2_ combined with ZIF‐67, and Cu(NO_3_)_2_ combined with Co(NO_3_)_2_, each at a total metal concentration of 200 μM.

The laccase‐like activity of Cu‐doped ZIF‐67 was measured by the colorimetric reaction of 2,4‐DP and 4‐aminoantipyrine (4‐AAP).^[27]^ Oxidized by laccase, 2,4‐DP reacts with coexisting 4‐AAP to form 4‐[(3‐chloro‐4‐oxo‐2,5‐cyclohexadien‐1‐ylidene)amino]‐1,2‐dihydro‐1,5‐dimethyl‐2‐phenyl‐3H‐pyrazol‐3‐one (CODAP), which has a maximum absorption at 510 nm, as shown in Scheme 1. Therefore, the increase in absorbance at 510 nm can be used to evaluate laccase activity. Cu50‐ZIF‐67 (16 mol % Cu) exhibited significant activity in the colorimetric reaction, while ZIF‐67 without copper ions showed no increase in absorbance at 510 nm, similar to ZIF‐8 (Figures 2 and 3a). Cu50‐ZIF‐8, containing both zinc and copper ions, also showed an increase in absorbance at 510 nm, similar to Cu50‐ZIF‐67. Despite containing more copper ions, Cu50‐ZIF‐8 (40 mol % Cu) exhibited lower activity than Cu50‐ZIF‐67 (16 mol % Cu), suggesting the utility of introducing copper into Co‐based ZIF‐67.

We also performed a colorimetric assay to assess the laccase‐like activity of Cu25‐ZIF‐67 (7 mol % Cu), which exhibited lower activity compared to Cu50‐ZIF‐67 (16 mol % Cu). This suggests that the presence of copper ions contributes to the increase in absorbance at 510 nm. Next, we varied the amounts of catalysts, Cu(NO_3_)_2_, Cu25‐ZIF‐67 (7 mol % Cu), Cu50‐ZIF‐67 (16 mol % Cu), Cu25‐ZIF‐8 (23 mol % Cu), and Cu50‐ZIF‐8 (40 mol % Cu), and plotted the rates of the colorimetric reaction measured at different copper ion concentrations, as shown in Figure 3b. The plot in Figure 3b shows that for the same amount of copper, Cu‐doped ZIF‐67 exhibited higher activity than Cu‐doped ZIF‐8 and Cu(NO_3_)_2_, whereas Cu‐doped ZIF‐8 showed activity comparable to Cu(NO_3_)_2_. In order to clarify the role of ZIF‐67 as an oxygen‐reducing catalyst, the colorimetric assay was conducted with Cu(NO_3_)_2_ in the presence of ZIF‐67 (Figure 3c). The solution containing Cu(NO_3_)_2_ and ZIF‐67 exhibited higher activity than Cu(NO_3_)_2_ alone. Additionally, no improvement in activity was observed when Co(NO_3_)_2_ was coexistent with Cu(NO_3_)_2_. These results clarify the unique role of ZIF‐67 in accelerating the redox cycle of Cu ions involving phenol oxidation. These results suggest that, as designed, Co‐based ZIF‐67 unit facilitates the reduction of oxygen, which is the half‐reaction in the laccase reaction.

**Figure 4:**
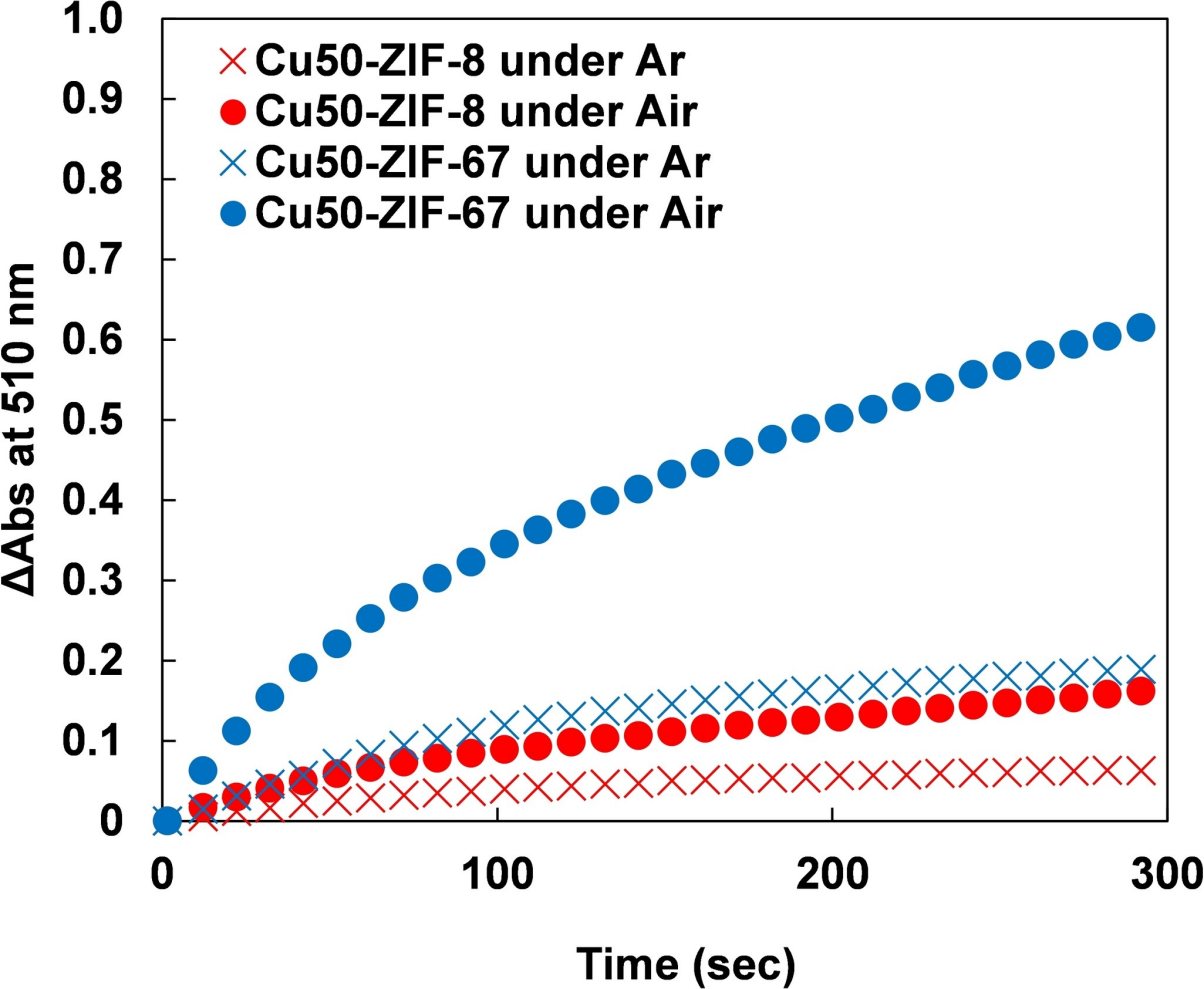
Time course of absorption intensity at 510 nm for the colorimetric assay using Cu50‐ZIF‐8 (red) or Cu50‐ZIF‐67 (blue) under air (circles) and argon (crosses), each at a metal concentration of 500 μM.

The oxidation of substrates by laccase requires oxygen. To determine if Cu‐doped ZIF‐67 also requires oxygen, the same colorimetric reactions were conducted under an argon atmosphere. Both Cu‐doped ZIF‐67 and Cu‐doped ZIF‐8 showed a decrease in reactivity under argon compared to atmospheric conditions (Figures 4 and Figure S4). The decrease in reactivity was more pronounced for Cu‐doped ZIF‐67 than for Cu‐doped ZIF‐8. These results suggest that Cu‐doped ZIF‐67 has higher reactivity with oxygen than Cu‐doped ZIF‐8, possibly due to cobalt ions in Cu‐doped ZIF‐67 facilitating oxygen reduction. Under argon, Cu‐doped ZIF‐8 exhibited a faster reaction rate than Cu‐doped ZIF‐67, which may be attributed to a higher number of copper ions in Cu‐doped ZIF‐8 compared to Cu‐doped ZIF‐67. Additionally, similar experiments were conducted using laccase at a copper concentration equivalent to 200 μM (Figure S5). The colorimetric reaction still proceeded while the activity under argon was lower than that under air. These findings suggest that Cu(II) ions in Cu‐doped ZIF‐67, Cu‐doped ZIF‐8, and laccase can still oxidize substrates under an argon atmosphere. However, oxygen's role in reoxidizing Cu(I) to Cu(II) is crucial for maximizing laccase activity.

**Figure 5:**
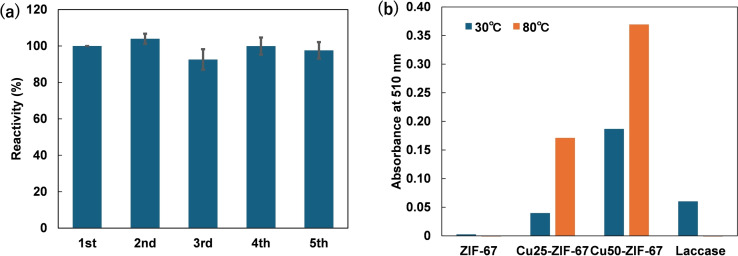
(a) Bar graph showing the reactivity for the colorimetric assay using Cu50‐ZIF‐67 (metal concentration: 500 μM, concentration of 2,4‐DP and 4‐AAP: 5 mM) during the recycling experiment up to the 5th cycle. Error bars represent data from three replicates. (b) Bar graph showing absorbance at 510 nm after 300 seconds for the colorimetric assay using ZIF‐67, Cu25‐ZIF‐67, Cu50‐ZIF‐67 (metal concentration: 200 μM) and laccase ([Cu]=200 μM) at 30 °C and 80 °C.

Upon two‐electron reduction, oxygen forms hydrogen peroxide, whereas upon four‐electron reduction, it becomes water. Laccase reduces oxygen by four electrons to produce water. It is essential to investigate whether Cu50‐ZIF‐67 reduces oxygen by four electrons or reduces it by two electrons to generate hydrogen peroxide. To elucidate this, an assay with horseradish peroxidase (HRP) and 2,2′‐azino‐bis(3‐ethylbenzothiazoline‐6‐sulfonic acid) (ABTS) was conducted. In this assay, the peroxidase HRP reacts even with low concentrations of hydrogen peroxide, oxidizing ABTS and producing a green ABTS cation radical. After one hour of the colorimetric laccase‐like activity assay using Cu50‐ZIF‐67 as a catalyst, we added HRP and ABTS but observed no green coloration indicative of ABTS cation radicals. Subsequently, we added hydrogen peroxide and observed immediate green coloration (Figure S6). These results suggest that Cu50‐ZIF‐67, similar to laccase, reduces oxygen via a four‐electron process, producing water rather than hydrogen peroxide during substrate oxidation. In order to investigate whether Cu ions leached from Cu‐ZIF‐67 during the reaction contributed to the activity, the supernatant of a Cu‐ZIF‐67 aqueous suspension was evaluated for the colorimetric assay. No colorimetric reaction was observed in the supernatant. These results indicate that Cu50‐ZIF‐67 is a unique artificial enzyme with phenol oxidation and oxygen reduction sites. The reaction activity remained almost constant even after recycling experiment up to the 5th cycle (Figure 5a). Interestingly, Cu50‐ZIF‐67 exhibited higher activity than laccase at 30 °C. XRD and SEM measurements of Cu50‐ZIF‐67 after the reaction indicate the material's structural stability under the given conditions, although an increase in surface roughness is observed (Figures S7 and S8). Additionally, ICP analysis showed that 0.6 % of the copper leached from Cu50‐ZIF‐67 after the reaction. Furthermore, at 80 °C, where laccase becomes completely inactivated, Cu50‐ZIF‐67 showed even higher activity than at 30 °C (Figure 5b).

**Figure 6:**
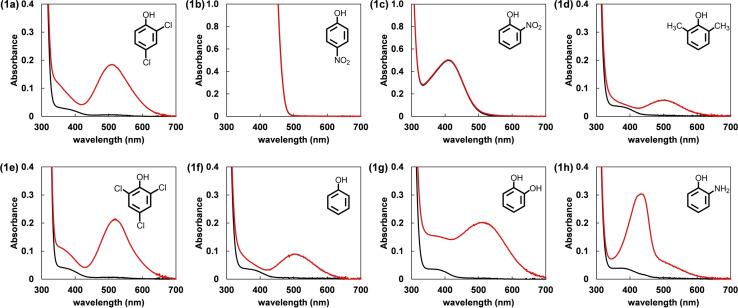
Absorption spectra of the colorimetric assay using 4‐AAP and **1 a**–**1 h**, and Cu50‐ZIF‐67 (metal concentration: 200 μM) as a catalyst just after (black line) and at 300 seconds (red line) after reaction initiation (red line) by adding each phenol substrate.

In many cases, the combination of 2,4‐DP (**1 a**) and 4‐AAP is used in the colorimetric assay of laccase activity. However, we investigated the reactions of phenols other than 2,4‐DP (**1 a**) with 4‐AAP, specifically **1 b**–**1 h** (Figure 6). similar absorption bands around 500 nm were observed when using **1 d**–**1 g** with 4‐AAP as substrates, similar to the case with **1 a** and 4‐AAP. When using **1 h** with 4‐AAP, an absorption band around 450 nm was observed. The absorption band around 450 nm was similar to that of phenoxazine, which is produced by the oxidative dimerization of **1 h**. Indeed, an increase in the absorption band around 450 nm was also observed when using **1 h** alone as a substrate. Therefore, it can be inferred that the oxidized form of **1 h** reacts with **1 h** itself much more efficiently than with 4‐AAP. In contrast, no changes were observed in the absorption spectrum when using 4‐nitrophenol (**1 b**) or 2‐nitrophenol (**1 c**) and 4‐AAP as substrates. The lack of reaction of **1 b** and **1 c** may be due to its high oxidation resistance.

## Conclusions

This study demonstrates that Cu‐doped ZIF‐67 (16 mol % Cu) exhibits significant laccase‐like activity, effectively oxidizing substrates and reducing oxygen through a four‐electron process to produce water rather than hydrogen peroxide. The copper sites within the ZIF‐67 framework mimic the mononuclear copper site of laccase, while the cobalt sites facilitate oxygen reduction. The incorporation of copper into ZIF‐67, although limited compared to ZIF‐8, enhances the material's catalytic activity, surpassing that of laccase, especially at elevated temperatures where laccase typically becomes inactivated.

The stability of Cu50‐ZIF‐67 across multiple reaction cycles and its resilience at high temperatures highlight its potential as a robust, reusable artificial enzyme. The absence of hydrogen peroxide production further validates its efficacy in mimicking laccase's natural electron transfer mechanisms.

Cu‐doped ZIF‐67 is a promising candidate for various industrial applications requiring high thermal stability and efficient catalytic activity, including bioremediation, dye decolorization, lignin modification, and biosensing. This work opens new avenues for developing laccase‐mimicking materials, potentially overcoming the natural enzyme's isolation, purification, and thermal stability limitations. Future research should focus on optimizing the incorporation of copper ions and further exploring other metal‐organic frameworks to enhance these artificial enzymes’ catalytic properties.

## Experimental Section

Reagents: All reagents, organic solvents, laccase from Trametes versicolor (Sigma‐Aldrich, 38429) and horseradish peroxidase (BBI solutions, HRP4) were purchased from Wako Pure Chemical Industries or Nacalai Tesque and used without further purification.

Physical Measurements: The morphology and metal distribution were observed using a Hitachi‐High‐Tech FE‐SEM SU8020 instrument equipped with energy‐dispersive X‐ray spectroscopy (EDS). Powder X‐ray diffraction (XRD) was conducted on a Rigaku SmartLab X‐ray diffractometer with a Cu‐*K*_α_ radiation (*λ*=1.54 Å) source (40 kV, 30 mA). Diffuse reflectance (DR) UV‐vis spectra were collected on a Shimadzu SolidSpec‐3700 DUV spectrophotometer. UV‐vis spectroscopy measurement for the kinetic assay was carried out using an Agilent 8453 UV‐visible Spectrometer. Contents of copper and zinc were analyzed on a Shimadzu ICPS‐8100 inductively coupled plasma atomic emission spectrometer (ICP‐AES).

Preparation of Cu‐doped ZIF‐67: Co‐only ZIF‐67 was synthesized as follows: Firstly, 328.0 mg (4 mmol) of 2‐methylimidazole (2‐MeIm) was dissolved in 10 mL methanol, resulting in solution A. Subsequently, a 10 mL mixture of a 100 mM solution of Co(NO_3_)_2_ in methanol was added dropwise to solution A, while keeping the mixture undisturbed in a thermostat at 50 °C. After a duration of 6 h, the resulting particles were collected by vacuum filtration using a membrane filter and subjected to vacuum drying. Similarly, Cu25‐ZIF‐67 and Cu50‐ZIF‐67 were synthesized using 10 mL methanol containing 25 mM Cu(NO_3_)_2_ and 75 mM Co(NO_3_)_2_ for Cu25‐ZIF‐67, and using 10 mL methanol containing 50 mM Cu(NO_3_)_2_ and 50 mM Co(NO_3_)_2_ for Cu50‐ZIF‐67. Cu50‐ZIF‐67 was synthesized using 16 mmol of 2‐MeIM.

Colorimetric assay of laccase activity: ZIF particles were dispersed in HEPES buffer (100 mM, pH 7.0) in a cuvette. After maintaining at 30 °C for 5 minutes, 4‐AAP and 2,4‐DP were sequentially added, resulting in final concentrations of 0.5 mM for both 4‐AAP and 2,4‐DP, with a total volume of 1.8 mL. The change in absorbance at 510 nm over time was measured. The same procedure was followed when using phenols other than 2,4‐DP as substrates.

Detection of hydrogen peroxide: The generation of hydrogen peroxide during the oxidation of 2,4‐DP by Cu‐doped ZIF‐67 was evaluated using HRP and ABTS. The reaction was conducted under the following conditions: 100 mM HEPES buffer at pH 7.0, 0.5 mM 2,4‐DP, and ZIF particles, incubated at 30 °C for 1 hour. Subsequently, HRP (0.1 μg/mL) and ABTS (1 mM) were added, and the change in absorbance at 414 nm, corresponding to the ABTS cation radical, was measured over time.

## Conflict of Interests

The authors declare no conflict of interest.

1

## Supporting information

As a service to our authors and readers, this journal provides supporting information supplied by the authors. Such materials are peer reviewed and may be re‐organized for online delivery, but are not copy‐edited or typeset. Technical support issues arising from supporting information (other than missing files) should be addressed to the authors.

Supporting Information

## Data Availability

The data that support the findings of this study are available from the corresponding author upon reasonable request.
