# Supplementary material for: Designing Artificial Laccase Catalysts by Introducing Substrate Oxidation Metals into Oxygen‐Reducing Metal‐Organic Frameworks: Cu‐Doped ZIF‐67
Source: Chemistry. 2024 Nov 16;30(72):e202402953. doi: 10.1002/chem.202402953 (PMC11665485; doi:10.1002/chem.202402953)
Supplement: Supplementary file 1 — Supporting Information [file CHEM-30-e202402953-s001.pdf]

Chemistry–A European Journal

Supporting Information

Designing Artificial Laccase Catalysts by Introducing Substrate Oxidation Metals into Oxygen-Reducing Metal-Organic Frameworks: Cu-Doped ZIF-67

Hiroki Nakahara and Yutaka Hitomi*

Supporting information

Designing Artificial Laccase Catalysts by Introducing Substrate Oxidation Metals into Oxygen-Reducing Metal-Organic Frameworks: Cu-doped ZIF-67

Hiroki Nakahara, and Yutaka Hitomi*

Department of Applied Chemistry, Graduate School of Science and Engineering

Doshisha University

1-3 Tatara Miyakodani, 610-0321 Kyotanabe, Kyoto (Japan)

E-mail: yhitomi@mail.doshisha.ac.jp

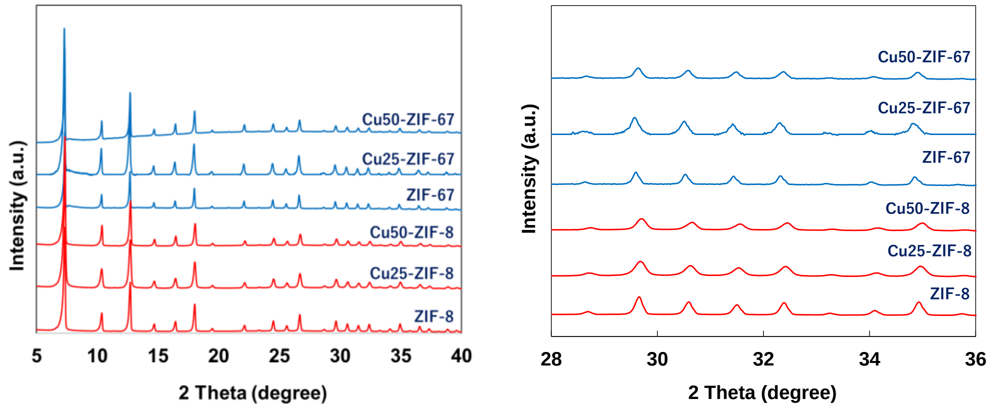

Figure S1. (a) powder XRD patterns of Cu50-ZIF-67 (Cu 16 mol%), Cu25-ZIF-67 (Cu 7 mol%), ZIF-67, Cu50-ZIF-8 (Cu 40 mol%), Cu25-ZIF-8 (Cu 23 mol%), and ZIF-8 and (b) powder XRD patterns expanding range from 28 to 36 degrees.

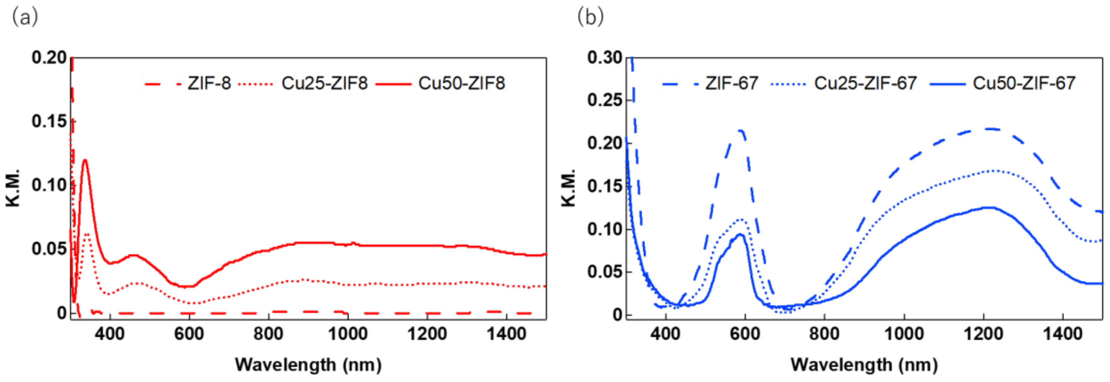

Figure S2. Diffuse reflectance UV-vis-NIR spectra of (a) Cu50-ZIF-8 (Cu 40 mol%), Cu25-ZIF-8 (Cu 23 mol%), and ZIF-8, each diluted 1/20 with $BaSO_4$, and (b) ZIF-67, Cu25-ZIF-67 (Cu 7 mol%) and Cu50-ZIF-67 (Cu 16 mol%), each diluted 1/50 with $BaSO_4$.

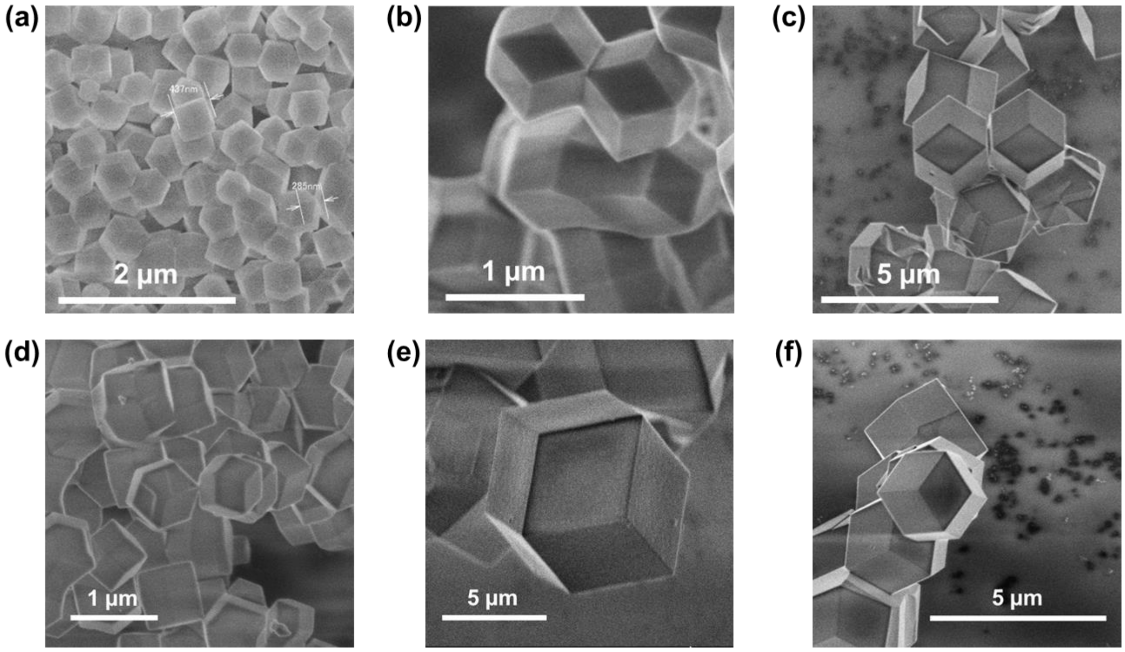

Figure S3. SEM images of (a) ZIF-8, (b) Cu25-ZIF-8, (c) Cu50-ZIF-8, (d) ZIF-67, (e) Cu25-ZIF-67, and (f) Cu50-ZIF-67.

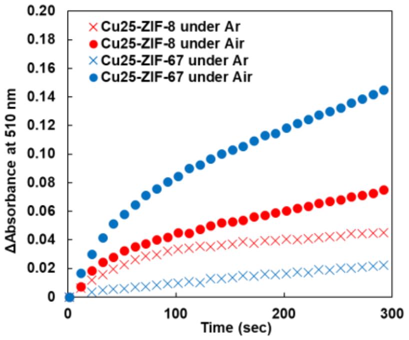

Figure S4. Time course of absorption intensity at 510 nm for the colorimetric assay using Cu25-ZIF-8 (red) or Cu25-ZIF-67 (blue) under air (circles) and argon (crosses), each at a metal concentration of 500 µM.

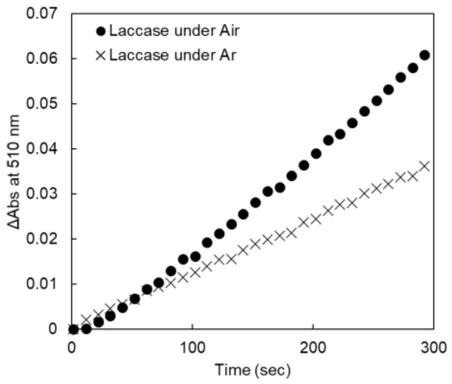

Figure S5. Time course of absorbance at 510 nm for the colorimetric assay with laccase under air (circles) and argon (crosses) conditions, at a copper concentration of 200 μM.

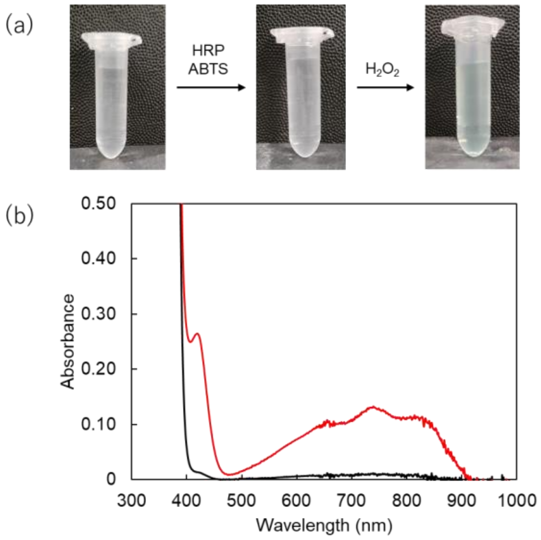

Figure S6. (a) Photographs of the solution after the reaction, the solution with HRP and ABTS added, and the solution with hydrogen peroxide, and (b) absorption spectra of the solution after the addition of HRP and ABTS (black line) and the solution after the subsequent addition of hydrogen peroxide (red line).

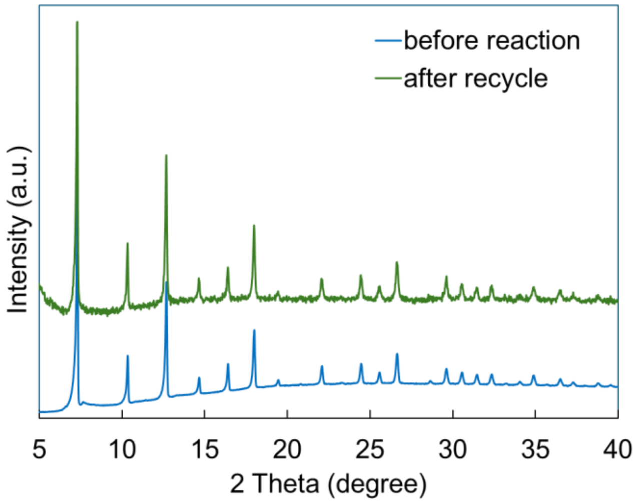

Figure S7. XRD patterns comparing Cu50-ZIF-67 before and after the reaction. The blue line represents the XRD pattern prior to the reaction, while the green line corresponds to the XRD pattern after the reaction.

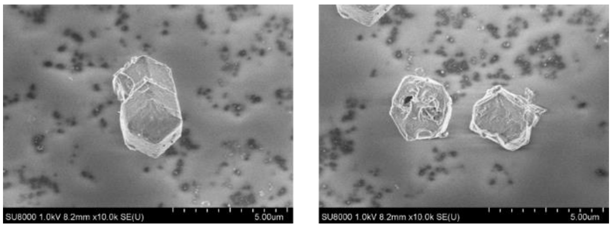

Figure S8. SEM images of Cu50-ZIF-67 after the reaction.
